# Warning Against Low-Density Lipoprotein Oxidation in Users of Oral Combined Contraceptives

**DOI:** 10.5935/abc.20180230

**Published:** 2018-12

**Authors:** Marcelo Chiara Bertolami

**Affiliations:** Instituto Dante Pazzanese de Cardiologia da Secretaria de Estado da Saúde de São Paulo, São Paulo, SP - Brazil

**Keywords:** Cardiovascular Diseases/mortality, Oxidation, Lipoproteins, LDL, Contraceptives, Oral, Stroke.

Cardiovascular diseases are the main cause of morbidity and mortality in the Western World and in our country.^1^ In the last years, this scenario has shown a decrease in the incidence of stroke, formerly the first cause of death.^2^ Today, its place has been taken by coronary heart disease.^2^ This change was due to better diagnosis and treatment of hypertension, the main cause of strokes, and the increase of the prevalence of risk factors for coronary heart disease such as obesity, diabetes, bad dietary habits, emotional stress and social deprivation, among others.^3^ Recently, an increase in myocardial infarction mortality, attributed to several causes, has been observed specifically among Brazilian^4^ and North-American^5^ young women. The article by dos Santos ACN et al.^6^ has focused on one of these possible causes. They studied low-density lipoprotein (LDL) oxidation in users of combined oral contraceptives, showing that this alteration of lipoproteins is increased in this group. LDL oxidation is considered one of the main participants in the atherosclerosis process development, as well as in its major clinical manifestations.^7^ They properly discussed the many possible causes of their findings and tried to establish correlations between LDL oxidation with many other variables. They referred to other studies that showed elevated C-Reactive Protein^8^ and blood pressure levels^9^ in users of combined oral contraceptives, which along with the known thrombogenicity of these agents (mainly in combination with tobacco smoking),^10^ can demonstrate the potential increase in cardiovascular risk in this group. The authors did not specify the types of oral contraceptives that were studied, which could be considered a study limitation. A practical consequence of the presented data is the fact that they are relevant for young women, who will need to find other kinds of contraception, such as IUDs, other oral contraceptives, and other possibilities to prevent the potentially deleterious effects of the combined oral contraceptives.
